# Details of hydrophobic entanglement between small molecules and Braun’s lipoprotein within the cavity of the bacterial chaperone LolA

**DOI:** 10.1038/s41598-019-40170-z

**Published:** 2019-03-06

**Authors:** Alister Boags, Firdaus Samsudin, Syma Khalid

**Affiliations:** University of Southampton, Southampton, SO17 1BJ United Kingdom

## Abstract

The cell envelope of Gram-negative bacteria is synthesized and maintained via mechanisms that are targets for development of novel antibiotics. Here we focus on the process of moving Braun’s lipoprotein (BLP) from the periplasmic space to the outer membrane of *E. coli*, via the LolA protein. In contrast to current thinking, we show that binding of multiple inhibitor molecules inside the hydrophobic cavity of LolA does not prevent subsequent binding of BLP inside the same cavity. Rather, based on our atomistic simulations we propose the theory that once inhibitors and BLP are bound inside the cavity of LolA, driven by hydrophobic interactions, they become entangled with each other. Our umbrella sampling calculations show that on the basis of energetics, it is more difficult to dislodge BLP from the cavity of LolA when it is uncomplexed compared to complexed with inhibitor. Thus the inhibitor reduces the affinity of BLP for the LolA cavity.

## Introduction

The cell envelope of Gram-negative bacteria is a complex chemical architecture composed of three distinct regions; the outer membrane, the inner membrane and the periplasmic space^1,2^. The latter is aqueous in nature, whereas the membranes are amphipathic: low dielectric hydrophobic cores surrounded by polar moieties on either side. All three regions contain proteins, which perform a variety of functions including maintaining the functional integrity of the cell envelope, and importantly, also synthesize the different molecules that constitute the cell envelope and localize them at the appropriate position within the whole architecture of the cell envelope^3^. This requires exquisite molecular choreography given the crowded environment of the cell envelope, and consequently a number of different pathways exist for synthesis and sorting of molecules.

Lipoproteins are abundant within the cell envelope, indeed Braun’s lipoprotein (BLP) is the most abundant protein in *E. coli*^4,5^. It is anchored in the outer membrane via a lipid moiety, which has three hydrocarbon tails at its N-terminus and is covalently bound to the cell wall peptidoglycan at its C-terminus^6,7^. Thus far it is the only known protein to be covalently bound to the cell wall. It provides stability to the cell envelope by linking the outer membrane and the cell well. Braun’s lipoprotein is synthesized at the inner membrane and then delivered to the outer membrane via a pathway involving the five Lol proteins, LolA, LolB, LolC, LolD, and LolE^8^. These proteins play key roles in the outer membrane-directed lipoprotein localization. The chaperone protein LolA has been shown to deliver BLP and other lipoproteins to the outer membrane-anchored protein LolB which then localizes them by a yet undetermined pathway^9–12^. The absence of LolA and LolB could result in toxic accumulation of mislocalized lipoproteins^13^. It has previously been shown that small hydrophobic molecules including MAC13243 (MAC) and its degradation products, S-(4-chlorobenzyl) isothiourea and 3,4-dichlorobenzyl carbamimidothioate, are able to bind to LolA and partially inhibit the protein *in vivo*^14,15^.

A previous study suggests that inhibitor molecules like MAC bind within the hydrophobic cavity of LolA and physically block access to BLP in a classic competitive inhibition manner^16^. Unable to bind, BLP therefore cannot be delivered to LolB and be correctly localized. Support for this theory comes from the observation that lipoproteins are partially retained at the inner membrane when *E. coli* strains are treated with MAC. However as yet there is no structure of either LolA nor LolB bound to BLP or to inhibitor molecules. The mode of BLP binding is therefore unknown, and important questions remain unanswered, for example how deep into the cavity of LolA does it bind, and how many acyl chains are accommodated in the cavity? Furthermore, there is no direct structural or molecular basis for the proposed mechanism of inhibition of LolA by blocking access to the hydrophobic cavity by small molecules. To address these questions, we have investigated the binding of BLP to LolA in the presence and absence of the inhibitor molecules, MAC and its degradation products, using atomistic molecular dynamics simulations. The first objective being to identify BLP binding modes and the second to identify the molecular processes that effect inhibition. Here, we propose a new molecular mechanism of inhibition of LolA by these small molecules. We find that both MAC and BLP bind LolA at the same time, whereby the lipid tails of the latter wrap around the former. Free energy calculations reveal that the MAC-BLP complex is easier to remove from LolA than BLP alone.

## Results

### Multiple binding modes of BLP to LolA

**Figure 1:**
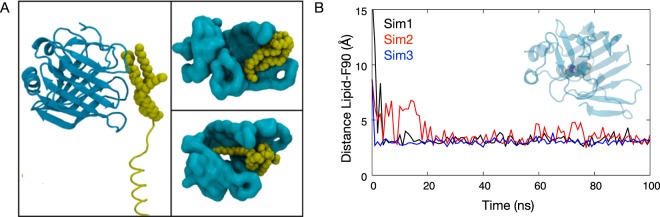
BLP binding modes within the hydrophobic cavity of LolA. (**A**) Left: the starting configuration, whereby BLP (yellow) is located just outside the LolA (cyan) cavity. Top right: binding mode in which three lipid tails of BLP bind near the mouth of the cavity. Bottom right: one lipid tail is deep within the cavity whereas the other two are bound at the mouth of the cavity. (**B**) Minimum distance between BLP lipid tails and residue F90 (shown in van der Waals representation in inset) from three independent simulations.

Firstly LolA and BLP were simulated in water and counterions by initiating the simulations with the BLP positioned just outside the hydrophobic cavity that is the proposed binding site of LolA^17^. The structure of LolA remained stable during the simulation as demonstrated by the similar RMSD progression compared to a simulation without BLP (Fig. S1). The main secondary structural motifs were also well preserved during these simulations (Fig. S2). We observed BLP moving into the cavity within 100 ns, after which the β sheet surrounding the cavity tilted to wrap around BLP, providing better interactions. The BLP lipid tails interacted with hydrophobic residues inside the binding cavity such as F90. There were 4 or 5 water molecules inside the cavity throughout the simulation; they were able to move in and out of the cavity even with BLP bound. We did not observe a single binding mode, but rather multiple LolA-BLP configurations such that at least one of the lipid tails of the lipoprotein was deep inside the cavity, stabilized by hydrophobic contacts (Fig. 1). In some simulations 2 or 3 lipid tails were inside the cavity. It is perfectly logical that binding within the cavity is general, as a single specific binding mode would likely restrict the LolA to chaperoning only BLP and not the other myriad lipoproteins within *E. coli*^12^. Furthermore, specific binding within LolA would likely stabilize the complex to an extent that would prohibit delivery of BLP to LolB. Indeed, it was previously shown that in the presence of LolB, a LolA-lipoprotein complex readily dissociated to form a LolB-lipoprotein complex, indicating a weaker, non-specific binding^11^.

### BLP binds to LolA in the presence of inhibitor molecules

Having established that 100 ns of simulation is sufficient to observe the movement of BLP to deep within the hydrophobic cavities of LolA, we next sought to characterize the effect of the inhibitors and to identify the mechanism of inhibition. Simulations were conducted in which 2 or 3 molecules of MAC were placed around the hydrophobic cavity of LolA with BLP placed just a bit further outside the cavity. Three independent 100 ns simulations were performed for each scenario. The rationale was to investigate (a) if the inhibitor would bind in the cavity and (b) if BLP could bind even if the inhibitor is present. From our previous simulations without the inhibitor, we identified residue F90 located in the LolA binding site to be one of the residues that interacted with BLP lipid tails; we therefore used a distance measurement between this residue and BLP as a metric to determine whether BLP binds to LolA.

**Figure 2:**
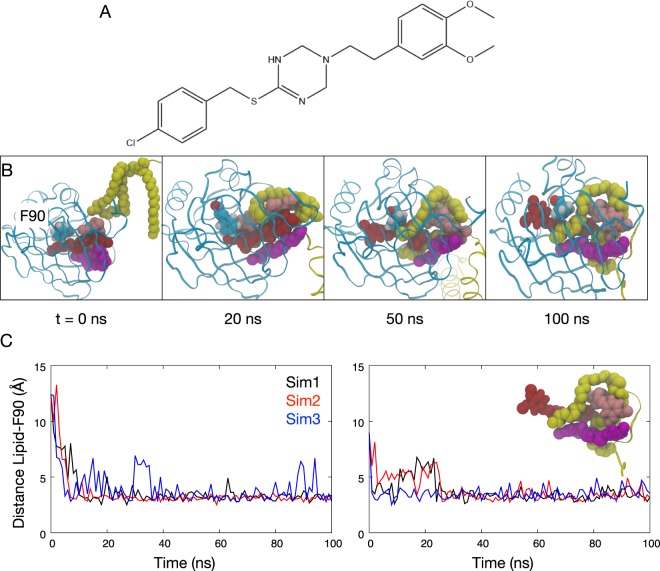
BLP binds to LolA even when two or three inhibitor molecules are present. (**A**) The chemical structure of MAC13243 inhibitor. (**B**) Snapshots at different time points during a simulation of LolA and BLP with three MAC molecules (red, pink, magenta) bound in the hydrophobic cavity. Residue F90 is labelled. (**C**) Minimum distance between the BLP lipids and F90 from simulations with two (left) and three (right) MAC inhibitors. Results are from three independent simulations. The insert illustrates hydrophobic entanglement of BLP lipids and three MAC molecules from one of the simulations.

Intriguingly even with 2 or 3 molecules of MAC within the cavity of LolA, the BLP lipid moiety can still bind inside the cavity, with the MAC molecules arranged around it (Fig. 2). Hydrophobic interactions stabilize the different molecules inside the cavity. The acyl chains of BLP are able to slide beyond the inhibitor molecules to reach deeper into the cavity. The acyl chains become entangled with the inhibitor molecules and the hydrophobic bulk of the inhibitor-BLP complex becomes wedged inside the cavity. We then replace the MAC molecules with its degradation products, S-(4-chlorobenzyl) isothiourea and 3,4-dichlorobenzyl carbamimidothioate, which are also expected to bind to LolA and inhibit BLP binding^15^. Similarly we observed binding of BLP to LolA in the presence of 2 or 3 molecules of the MAC degradation products within 100 ns (Fig. S3), further corroborating our hypothesis that the binding of these hydrophobic inhibitors does not occlude lipoprotein binding to LolA.

**Figure 3:**
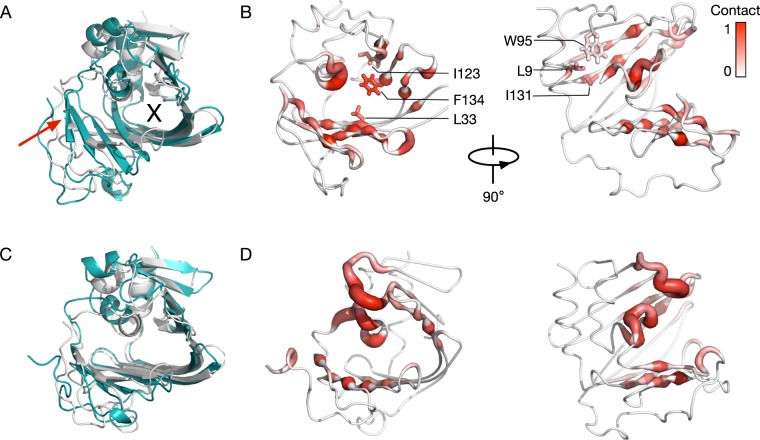
LolA binding site conforms to the shape of BLP lipid. (**A**) Structural comparison of LolA at the end of a simulation with zero MAC (cyan) to the crystal structure (grey). The tilt of the β-sheet is highlighted by the red arrow and the binding cavity is marked “X”. (**B**) Contact analysis with a distance cut-off of 4 A performed for each residue of LolA with BLP lipid averaged over three simulations. A score of 1 indicates contact made throughout the entire simulations. Residues that show more contact in simulations with zero MAC compared to that with three MACs are labelled. (**C**,**D**) Same analyses as (**A**,**B**), respectively, performed for simulations with 3 MACs.

Our results are in contrast to the suggestions in the literature that binding of these molecules inside the hydrophobic cavity prevents binding of BLP;^16^ in other words our simulations show that these molecules are not conventional competitive inhibitors, but instead uncompetitive inhibitor. It is worth noting that a system with two MAC molecules translates to a concentration of MAC of around 750 µg/ml, which is much higher than its minimum inhibitory concentration (16 µg/ml)^14^, and even at this higher concentration binding of BLP was observed.

### Inhibitor molecules reduce BLP interactions with LolA

To elucidate the mechanism of inhibition of MACs and its degradation products we looked for any structural differences between the LolA-BLP complexes in the absence and presence of MACs. BLP binding to LolA was found to elicit a larger conformational change of the latter in the absence of MAC, compared to when MACs were also bound. There was a pronounced tilt of the β sheets, particularly involving strands β3-β6, which allowed the binding site to ‘wrap around’ the BLP lipid tails (Fig. 3A). We performed principal component analysis and found the motion along the first eigenvector to involve the β sheets tilting towards the position of the BLP lipid tails (Fig. S4). In contrast this conformational change was absent in the simulations in which MACs were bound in the LolA cavity (Fig. 3C). LolA retained its original structure throughout in these simulations. This explains the lower RMSD progression from the X-ray structure in these simulations compared to those in which MAC was absent (Fig. S1).

The conformational change that occurred when BLP alone is bound to LolA resulted in an increased number of interactions between the BLP lipids and residues in the LolA binding site. Contact analysis using a distance cutoff of 4 Å revealed that in the absence of MACs the BLP lipid tails interacted with more hydrophobic residues located deep within the binding cavity such as L9, I23, W95 and I131 (Fig. 3B). On the other hand, when MACs were present the BLP lipid moieties contacted residues that were mostly found around the opening of the binding site (Fig. 3D). The results of the conformational and contact analyses together suggest that the MAC-BLP acyl chain entanglement in the LolA binding site restricts conformational changes of LolA that are necessary for BLP interactions with key hydrophobic residues, and therefore results in shallower and potentially weaker binding.

While the BLP lipid tails occupy the LolA binding site, the BLP helix was found to bind to the outer solvent-exposed surface of LolA. To understand the conformational dynamics experienced by this helix during the simulations with and without MACs, we performed cluster analysis using the algorithm of Daura *et al*.^18^. Each conformation is assigned to a cluster based on an RMSD cutoff of 2 Å. The cluster size as well as the representative structures of the most frequently sampled conformation are shown in Fig. S5. Comparing two independent repeat simulations for each system we found the BLP helix to show very different dynamics: for example, in one simulation with 3 MACs the helix mostly sampled an elongated conformation, whilst in another simulation it preferred an L-shape conformation. We note that this suggests that our 100 ns duration simulations may not have converged vis-à-vis the conformational dynamics of the BLP helix and longer simulations are therefore needed to study the dynamics of the full BLP protein with LolA, however this is not the focus of the present study.

### Inhibitor molecules weaken LolA-BLP binding

**Figure 4:**
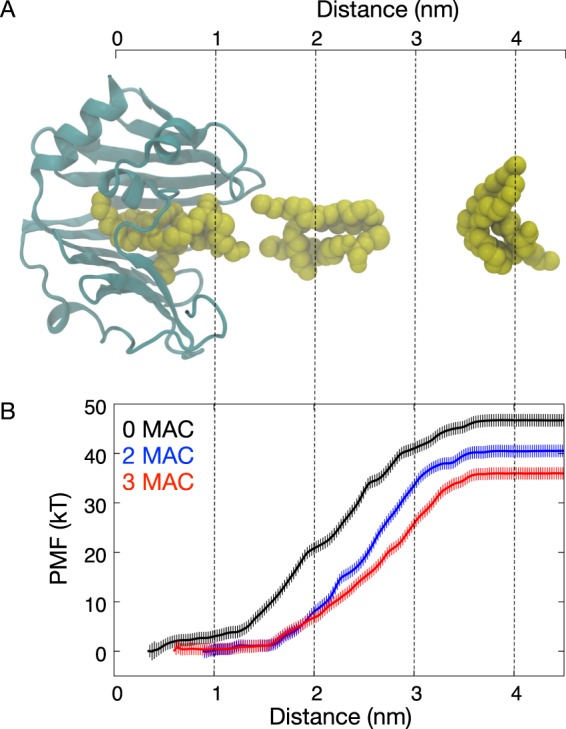
Free energy profiles of BLP lipid unbinding from LolA. (**A**) Snapshots taken from a steered MD simulation whereby BLP lipid is pulled out from LolA binding site. The reaction coordinate is the distance between centers of mass of the Sulphur atom on BLP lipid and the protein. (**B**) PMF profiles for LolA unbinding from umbrella sampling calculations. Error bars indicate standard deviations estimated from bootstrapping.

To quantify the strength of BLP binding to LolA in the absence and presence of 2 or 3 MACs, we performed a potential of mean force (PMF) calculation along the dissociation pathway of BLP. Our cluster analysis shows that long-timescale simulations are required to adequately sample the conformational dynamics of the BLP helix when it binds to the LolA outer surface; we therefore removed the helix for our PMF calculation to help achieve convergence. We first performed a steered MD simulation to pull the bound BLP lipid into solution (Fig. 4A), and subsequently used snapshots from this simulation as windows for a series of 100 ns umbrella sampling MD simulations. We achieved adequate sampling along the reaction coordinate as indicated by the histogram overlap (Fig. S6). PMF profiles constructed from increasing amount of simulation time suggest that convergence was reached after around 30 ns (Fig. S7).

Our PMF profiles shows that the BLP lipid bound most strongly to LolA when there was no MAC present, with the free energy of dissociation estimated to be around 45 kT (Fig. 4B). In the presence of two and three MACs these free energy values are reduced to around 40 kT and 37 kT respectively. For the systems with zero and three MACs, two additional PMF profiles were constructed using independent sets of steered and umbrella sampling MD from different starting coordinates. Encouragingly we found all three repeats to agree with each other within statistical errors and point towards the higher binding affinity of BLP lipid in the absence of MAC inhibitors (Fig. S8). This is concordant with our contact analysis, which shows that in the absence of MACs more interactions were formed between the BLP lipid moieties and hydrophobic residues in the LolA binding site. These additional interactions with residues found deep within the binding cavity therefore are crucial for a strong BLP binding. It is worth noting that even in the presence of MAC inhibitors binding is still likely, albeit weaker, given the positive value of the free energy of dissociation. Again, this suggests that MAC and potentially its degradation products are not competitive inhibitors of LolA, but rather reduce the binding affinities of BLP.

### Inhibitor molecules diffuse freely across the cell wall

**Figure 5:**
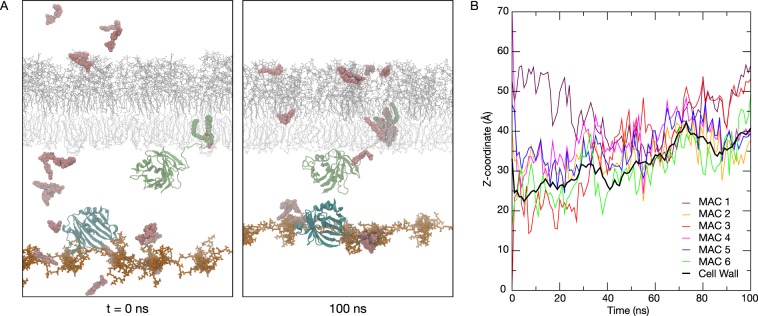
Simulation of MAC inhibitors with LolA and LolB. (**A**) Snapshots taken at the beginning (left) and at the end (right) of a simulation with LolA (cyan), LolB (green) embedded in an outer membrane model (grey), cell wall (orange), and 20 MAC molecules (pink). (**B**) The Z-coordinates of the centers of mass of six different MACs with respect to that of the cell wall throughout a 100 ns simulation, to highlight the ability of the inhibitor to diffuse through the cell wall layer.

To put our study in the larger context of the bacterial cell envelope, we then built a system incorporating the outer membrane^19^ with LolB embedded, a single layer of peptidoglycan cell wall^20,21^, and LolA positioned on the cell wall on the outer membrane side of the periplasm. Twenty MAC molecules were placed randomly in the solvent at the beginning of the simulation. We performed three independent 100 ns simulations to study how the MACs behave in the cell envelope environment and whether they readily bind to either LolA and LolB. Unsurprisingly we found most MACs spontaneously inserted into the hydrophobic core of the outer membrane (Fig. 5A). We did not observe MAC binding to either LolA or LolB within the 100 ns timescale of the simulation, which is largely a consequence of the greater distance between the MACs and the proteins here than in the simulations described above. More crucially several MAC molecules were observed to diffuse freely across the cell wall (Fig. 5B). This suggests that the inhibitor molecules can bind LolA even when this protein is located on the inner membrane side of the periplasm.

## Discussion

In summary, we have shown that the lipid moieties of BLP binds deep into the hydrophobic cavity of LolA via non-specific binding modes. The small molecule, MAC13243 and its degradation products readily bind into the same cavity of LolA. We have shown that BLP lipid tails are able to bind into the cavity even when the cavity is already occupied by 3 MAC molecules, albeit not as deeply into the cavity as when the small molecules are absent. Inside the cavity, the BLP tails and MAC molecules become entangled with each other. Interestingly LolA undergoes a conformational change when BLP alone binds into the cavity, this provides additional stabilizing interactions between BLP and LolA. Free energy calculations reveal that dissociation of the BLP-inhibitor complex from LolA is energetically more favorable than dissociation of BLP alone from LolA. Based on these observations we propose that MAC and its degradation products inhibit LolA by reducing the binding affinity of BLP for LolA. It has been shown that in the presence of LolB (the protein to which BLP is delivered by LolA), the LolA-BLP complex dissociates, indicating that the affinity of BLP for LolB is higher than its affinity for LolA^11^. It is possible that MAC reduces the LolA-BLP affinity such that the MAC-BLP complex can become dislodged from LolA before reaching BLP. We would like to state here that while the free energy differences come directly from our simulations, the hypothesis regarding inhibition mechanism is not a conclusion based on our own results, but some thoughts on the implications of the results, which need further work (experimental and simulation) to confirm.

## Methods

### The models

The BLP monomer was constructed from the X-ray the structure reported by Shu *et al*. (PDB: 1EQ. 7)^7^. The N-terminus was attached to the tripalmitoyl-S-glyceryl-cysteine residue as previously used in our simulations of BLP and OmpA^21^. The structures of LolA and LolB were obtained from the protein database (PDB: 1IWL and 1IWM respectively)^17^. The first nine amino acids of LolB were added in using Modeller 9.19^22^ and the modelled protein was functionalised with a tripalmitoyl-S-glyceryl-cysteine residue (same lipid moiety as for BLP). MAC13243 and its degradation products, S-(4-chlorobenzyl) isothiourea and 3,4-dichlorobenzyl carbamimidothioate, were parameterised using the Automated Topology Builder (ATB)^23^ with parameters for the GROMOS 54A7 forcefield. The outer membrane model comprises Ra LPS lipids of the R1 core type^24,25^, in the upper leaflet, whilst the lower leaflet is composed of the following phospholipids: 90% 1-palmitoyl 2-cis-vaccenic phosphatidylethanolamine, 5% 1-palmitoyl 2-cis-vaccenic phosphatidylglycerol and 5% 1-palmitoyl 2-cis-vaccenic 3-palmitoyl 4-cis-vaccenic diphosphatidylglycerol)^26–29^. This model of the OM has previously been used and validated in our simulation studies^20,21,30,31^.

### Atomistic MD simulations

All simulations were performed using the GROMACS 2018 code^32^, the GROMOS 54A7 force field with the SPC water model^33^. Each simulation was run for 100 ns and three independent repeats were performed. The temperature was maintained at 310 K using the velocity rescale thermostat with a time constant of 1 ps^34^. The pressure was maintained isotropically in simulations that did not contain a membrane, and semi-isotropically in membrane-containing simulations, at 1 atm using the Parrinello-Rahman barostat with a time constant of 1 ps^35^. All bonds were constrained using the LINCS algorithm to allow for an integration time step of 2 fs^36^. Long-range electrostatics were described using the particle mesh Ewald method^37^. The long-range electrostatic cut-off was 1.4 nm and the short-range van der Waals cut-off was also 1.4 nm. All systems were charge neutral. The LPS molecules were neutralised by Ca^2+^ ions, and the simulation systems contained additional 0.2 M NaCl ions. The trajectories were visualised in VMD^38^ and analyses were performed using in-house scripts and GROMACS tools.

### PMF calculation

Steered and umbrella sampling MD simulations were performed to generate inputs for the calculation of PMFs along a reaction coordinate parallel to the BLP dissociation pathway from LolA binding site. Snapshots at the end of the 100 ns simulations described above were used as starting configurations. For systems with zero and three MACs three independent sets of steered and umbrella sampling MD simulations were performed starting from three different snapshots from the equilibrium simulations. To aid convergence the BLP helix was removed in all systems leaving only the lipid moieties in the LolA binding site. A short 1 ns simulation was performed to re-equilibrate each system. Constant-velocity (0.1 nm ns^−1^) steered MD simulation was performed to pull the BLP lipid away from the binding site along the y-axis using an elastic spring (force constant of 100 kJ mol^−1^ nm^2^) applied to the Sulphur atom on its headgroup. The backbone atoms of LolA was positionally restrained with a force constant of 1000 kJ mol^−1^ nm^2^ during this simulation. From this trajectory 50 umbrella sampling windows were then selected based on the distance between the centers of mass of the Sulphur atom and LolA along the reaction coordinate with a separation of 1 Å between windows. For each window a 100 ns simulation was performed with the center of mass of the Sulphur atom restrained in the vector of the reaction coordinate using a harmonic force constant of 1000 kJ mol^−1^ nm^2^. No restraint was imposed on LolA. The weighted histogram analysis method (WHAM)^39^ incorporated in the GROMACS *gmx wham* tool^40^ was used to compute the PMF from the umbrella sampling data. Histogram overlaps were plotted (Fig. S6A), and extra sampling windows were performed at missing coordinates to ensure adequate sampling. Integrated autocorrelation time (IACT) was computed for each umbrella sampling window (Fig. S6B); as IACTs computation is potentially inaccurate due to limited sampling, the IACTs along the reaction coordinate were subsequently smoothed with a Gaussian filter as described in Hub *et al*.^40^. The PMF profiles were then generated by WHAM taking these IACTs into account. Statistical errors were estimated using bootstrap analysis whereby new random trajectories were generated with data points distributed according to the given histograms and properly autocorrelated based on the previously computed IACT values. For each PMF profile, 100 bootstrap trials were performed. To confirm that the PMF has converged within the 100 ns umbrella sampling simulations, PMF profiles were constructed from increasing amount of simulation time (Fig. S7). We found in all systems and repeats PMF profiles converged after around 30 ns; the first 30 ns of the simulations was therefore regarded as equilibration time and excluded from the final PMF calculations.

## Supplementary information

Supplementary information
