# Supplementary material for: Details of hydrophobic entanglement between small molecules and Braun’s lipoprotein within the cavity of the bacterial chaperone LolA
Source: Sci Rep. 2019 Mar 6;9:3717. doi: 10.1038/s41598-019-40170-z (PMC6403396; doi:10.1038/s41598-019-40170-z)
Supplement: Supplementary file 1 — Supplementary information [file 41598_2019_40170_MOESM1_ESM.docx]

Details of hydrophobic entanglement between small molecules and Braun’s lipoprotein within the cavity of the bacterial chaperone LolA.

Alister Boags^1*^, Firdaus Samsudin^1*^ and Syma Khalid^1**^

^1^University of Southampton, Southampton, SO17 1BJ, United Kingdom.

*Authors contributed equally

**Corresponding author: [S.Khalid@soton.ac.uk](mailto:S.Khalid@soton.ac.uk)

Figure S1: All-atom RMSD of LolA throughout 100 ns simulations without BLP, with BLP plus zero, two, or three MACs. Data shown from two independent repeats.

Figure S2: Secondary structure analysis of LolA. The percentage of residues in LolA forming (A) β-sheet, and (B) ɑ-helix throughout the simulations for apo structure and BLP bound with either zero, two or three MACs. (C) The preservation of secondary structure motif in LolA during these simulations. β-sheet is red, ɑ-helix is blue, bend is green, turn is yellow, and unstructured coil is white. This is taken from one of the apo simulations of LolA. (D) Same analysis for one of the simulations of LolA with BLP bound without MAC. (E) Same analysis for one of the simulations of LolA with BLP bound with 3 MACs.

Figure S3: Simulations of LolA with BLP in the presence of the degradation products of MACs. (A) The chemical structure of S-(4-chlorobenzyl) isothiourea (left) and a snapshot at the end of one of the simulations, whereby LolA is cyan, BLP lipid is yellow, and S-(4-chlorobenzyl) isothiourea molecules are red, pink and magenta (right). (B) Minimum distance between BLP lipid and residue F90 from simulations with two (left) and three (right) S-(4-chlorobenzyl) isothiourea molecules. (C) The chemical structure of 3,4-dichlorobenzyl carbamimidothioate (left) and a snapshot from the end of a simulation (right). (D) Minimum distance analysis (as described in (B) for S-(4-chlorobenzyl) isothiourea molecules.

Figure S4: Principal component analysis was performed on the structure of LolA from a simulation of LolA with BLP (without MACs). The figure shows an overlay of LolA structures to show the motion along the first eigenvector. The arrows indicate the tilting of the β-sheet during the 100 ns simulation as LolA adapted to the BLP lipid tails.

Figure S5: Cluster analysis of the BLP helix during the 100 ns simulations with BLP in the presence of (A) zero MAC, (B) two MACs, and (C) three MACs. The graphs show the size of each cluster ordered from the most frequently sampled conformation to the least frequently sampled. Data are taken from two independent repeats. Inset shows representative structures of the most dominant structures.

Figure S6: (A) Histogram overlap from umbrella sampling simulations of systems with zero (top), two (middle), and three MACs (bottom). (B) Integrated autocorrelation time (IACT) estimated for each window by direct integration of the autocorrelation functions (black circles) and subsequently smoothed by a Gaussian filter (red circles).

Figure S7: PMF profiles generated from increasing amount of simulation time. The figure on the left shows the PMF for the entire reaction coordinate, whilst the figure on the right shows an enlarged image indicated by the dotted box, with statistical errors estimated using bootstrapping.

Figure S8: Three PMF profiles were calculated for the systems with zero and three MACs using three independent sets of starting configurations, steered MD and umbrella sampling windows. Error bars show statistical uncertainties calculated using bootstrap analysis.
